# The French Affective Images of Climate Change (FAICC): A Dataset With Relevance and Affective Ratings

**DOI:** 10.3389/fpsyg.2021.650650

**Published:** 2021-08-06

**Authors:** Sarah Ottavi, Sébastien Roussel, Arielle Syssau

**Affiliations:** ^1^EPSYLON, Univ. Montpellier, Univ. Paul Valéry Montpellier 3, Montpellier, France; ^2^CEE-M, Univ. Montpellier and CNRS, INRAE, Institut Agro, Univ. Paul Valéry Montpellier 3, Montpellier, France

**Keywords:** climate change, emotion, French version, image database, valence, arousal, relevance ratings

## Introduction

*Climate change is not only a scientific phenomenon, but also a cultural one*. (Lehman et al., 2019).

Imagery related to climate change (CC) has been widely used for communication, research, and even advertising (Trumbo, 1999; Moser, 2010; Pezzullo and Cox, 2017). As the representation of CC seems to vary from one individual to another, imagery is very important to ensure that messages concerning CC are effectively communicated by experts, especially in a society where visual images predominate in all forms of media and communication (Nicholson-Cole, 2005; Lehman et al., 2019). Indeed, Leiserowitz (2006) argues that Americans' stated responses to questions about CC are strongly influenced by imagery, combined with affect and values. However, although CC communication should “address cognition, affect, and behavior, without reinforcing a sense of fatalism and disengagement” (Manzo, 2010), to our best knowledge no study has looked at the level of relevance to CC of the imagery used, as well as its emotional value within a population, except the Lehman et al.'s 2019 original database, which is American-based.

In this paper, we provide a data report that describes an original dataset named French Affective Images of Climate Change (FAICC) database. The main objective is to provide tools for CC assessment. Images are rated by a sample of non-experts according to three variables: relevance to CC, arousal, and emotional valence. The database provides for each image an identification number, the mean rating and standard deviation of ratings for relevance, arousal and valence, respectively.

## Data Collection

### Experimental Procedure and Participants

The experiment was conducted at the Université Paul Valéry Montpellier 3 (UPVM3) (France) during sessions organized in January 2020 and February 2020, respectively. 106 participants (63 women and 43 men) ranging in age from 18 to 29 years old (*M* = 20.24, *SD* = 2.572) were recruited randomly from psychology classes, receiving course credits for their participation. The experiment was single-blind and lasted about 45 mins.

### Image-Rating Stimuli

204 images were gathered from Google searches. We used the French translations of terms used by Lehman et al. (2019): (1) “*changement climatique*” (climate change), (2) “*causes du changement climatique*” (climate change causes), (3) “*impacts du changement climatique*” (climate change impacts), and (4) “*solutions au changement climatique*” (climate change solutions), and we selected the top images—these are the images that came up first in our google searches according to our selection criteria, with a satisfactory format and royalty free—, resulting in 237 images. Some images belonged to different categories and appeared in different searches, so we removed redundancies (*n* = 33), then resulting in 204 images.

Since Google searches consider the geographical location of the searches (Cooper et al., In press), we did not find any common images with Lehman et al.'s 2019 database. As a consequence, in order to examine the external validity of our ratings, 100 images were added from Lehman et al.'s 2019 database: the 32 most and the 32 least relevant images to CC with regards to the average relevance ratings, as well as 36 images randomly selected which were neither relevant nor irrelevant to CC.

The overall 304 images were divided into two sets of images to ensure individuals' rating does not require too much cognitive effort while conducting evaluation sessions that were no longer than 60 minutes. This division was made ensuring that there were as many images on the same theme in both sets (e.g., as many images with a blue sky/with a cloudy sky/etc.). Consequently, each set contained 150 unique images [of which, 50 images from Lehman et al.'s 2019 database in each set], 1 common that was repeated to assess the reliability of the ratings, and 3 common ones dedicated to the practice session.

301 images were then rated by the participants, i.e., 150 ^*^ 2 (per set) plus 1 (common one for reliability)—leaving aside the pictures used for the practice session.

### Image-Rating Task

The image-rating task was designed using OpenSesame software (version 3.2.5 -*Kafkaesque Koffka*- Mathôt et al., 2012) and followed exactly the same procedure as Lehman et al. (2019). Each participant was individually seated in front of a computer in a laboratory room. After filling out a consent form, participants were told that they were going to be shown images on the computer screen and were instructed to rate their personal reaction with regards to each image. Images were displayed above a question and a 9-point Likert scale (rating scale of 1–9): participants were asked how irrelevant or relevant each image was to CC (relevance), how calming or exciting it made them feel (arousal), and how the emotion evoked by the image is rather negative (unpleasant) or positive (pleasant) (valence), consistently in that order. Each image was appraised for each of the three variables before the next image was displayed, in a random order for each participant—relevance scale: 1. not relevant/9. relevant; arousal scale: 1. calm/9. excited; valence scale: 1. negative/9. positive. Participants used the numeric keypad on the computer keyboard to assign scores.

Prior to the effective rating-task, the session began with a practice session in using the 3 common pictures to ensure that participants had a good understanding of the task functionalities. Time was not limited but was recorded.

## Descriptive Statistics for the Variables Measured

### Descriptive Statistics

The descriptive statistical analysis is based on the 201 original images from our searches, leaving the 3 common ones dedicated to the practice session as well as the 100 images from Lehman et al.'s 2019 database aside. The mean rating of each variable (relevance, arousal, and valence) was collected for each image, and we present in Table 1 images distribution (share) according to relevance to CC and emotional valence ratings (<4; 4–6; > 6). Pearson's correlations were performed to assess the relationships between (A) relevance and arousal, (B) valence and relevance, and (C) valence and arousal.

**Table 1:** Summary of images distribution (share) according to relevance and valence ratings.

		**Valence levels**			**Total**
		**Negative (<4)**	**Neutral (4–6)**	**Positive (>6)**	
**Relevance levels**	**Low (<4)**	–	5 (2.49 %)	1 (0.5%)	6 (2.98%)
	**Medium (4–6)**	10 (4.98 %)	32 (15.92%)	12 (5.97%)	54 (26.87%)
	**High (>6)**	106 (52.73 %)	28 (13.93%)	7 (3.48%)	141 (70.15%)
**Total**		116 (57.71 %)	65 (32.34%)	20 (9.95%)	201 (100%)

**Figure 1:**
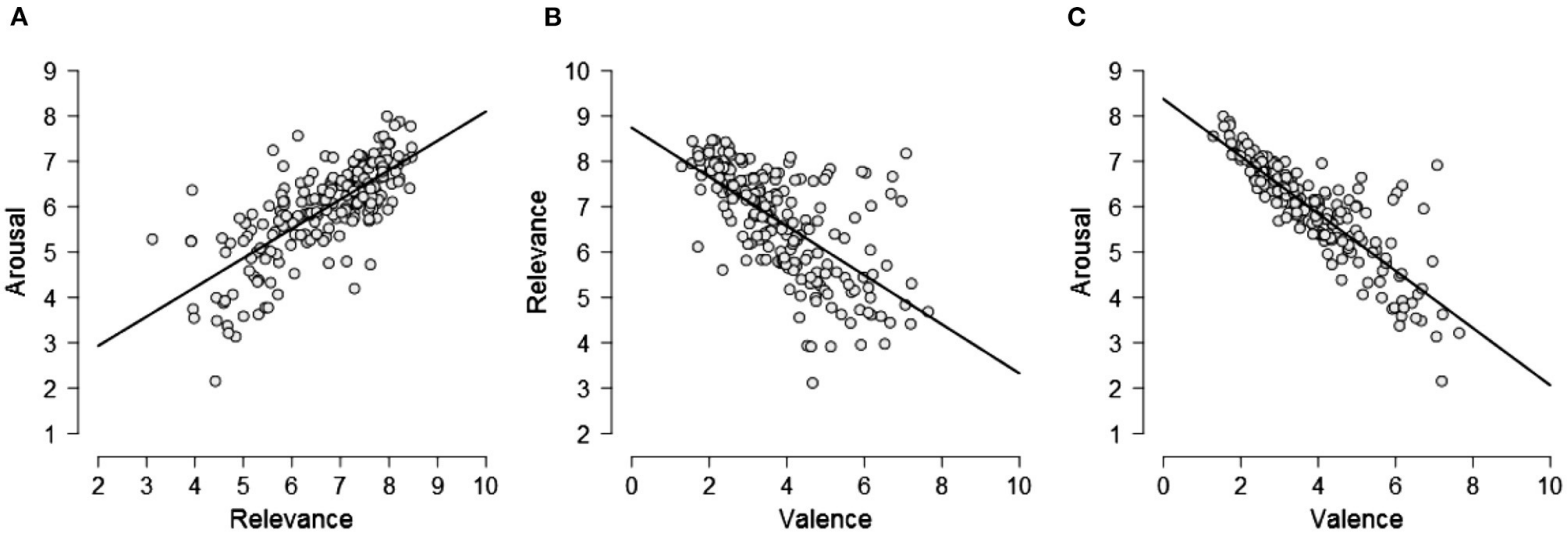
**(A)** There was a strong positive correlation between images that were rated high in relevance to CC and images that were rated high in arousal. **(B)** There was a strong negative correlation between the images rated low in valence and the images rated high in relevance to CC. **(C)** There was also a strong negative correlation between the images rated low in valence and the images rated high in arousal.

Preliminary results show that the means relevance scores of the 201 images (*M* = 6.653, *SD* = 1.169) were positively correlated with the means image arousal ratings (*M* = 5.941, *SD* = 1.032), *r* = 0.731, *p* < 0.001. The correlation indicates that the images assessed as most relevant to CC were also assessed as very exciting (Figure 1A). The mean relevance ratings also displayed a negative correlation with the mean valence ratings (*M* = 3.867, *SD* = 1.389), *r* = −0.644, *p* < 0.001. The correlation shows that the images rated as highly relevant to CC tended to also be rated as low valence (i.e., very negative) (Figure 1B). There was also a strong negative correlation between the mean arousal scores and the mean valence scores of the images, *r* = −0.849, *p* < 0.001, showing that the images rated as most exciting were also among the most negative (Figure 1C).

### Intra-Rater Reliability

As each participant's image list contained a repeated image, intra-rater reliability was assessed by examining the consistency with which they rated this repeated image for each of the variables (relevance, arousal, and valence). Raters' evaluation seemed to be consistent for all variables with no significant statistical difference in ratings [relevance: *t*(210) = 1.817, *p* = 0.071; arousal: *t*(210) = −0.898, *p* = 0.370; valence: *t*(210) = −0.43, *p* = 0.668].

### External Validity

In order to examine the external validity of our ratings, we now focus on the correlations with the ratings for the 100 images used from Lehman et al.'s 2019 database. Pearson's correlations were performed and showed that our relevance ratings for the images and those of Lehman et al. (2019) seemed to be highly positively correlated, *r* = 0.888, *p* < 0.001, as are the average valence ratings, *r* = 0.923, *p* < 0.001, which means that the ratings reported within our French student population very slightly differ from those reported within the American student population in Lehman et al. (2019). The arousal average assessment is, to a lesser extent, also positively correlated *r* = 0.433, *p* < 0.001. This distinction is consistent with previous research showing greater variability for arousal than for valence ratings (Eilola and Havelka, 2010; Monnier and Syssau, 2014). To conclude, these preliminary results show a potential good external validity.

## Possible Use and Continuation

The FAICC database provides ongoing research material for researchers who are interested in imagery and its implications for CC. Some of the possible uses of a set of emotional images relevant to climate change are, for example, advertising or scientific research, in which this material can be used to observe the impact of emotional images on attitudes or even a commitment to the environmental cause. The FAICC database is relevant in the French context, and by extension at the European-scale. This material could be used in comparative studies and cross-cultural studies. Along with a cultural distinction analysis in ratings, it may be interesting to investigate other cultural features (e.g., high-income vs. middle- and low-income countries, or natural hazards exposure of the populations), as well as possible generational distinctions in the visual perception of climate change.

## Data Availability Statement

The dataset for this study can be found online at the FAICC repository. i.e., https://upvdrive.univ-montp3.fr/index.php/s/5M8EDHwS2L6Tsa4.

## Ethics Statement

This study was carried out in accordance with the recommendations of the deontological code of psychology (adopted in 1996 by French academic societies, and then by 28 French non-academic organizations of psychologists; updated in 2012). All participants provided written informed consent to participate in the study. Participants were informed that, in accordance with the Informatique et libertés law of January 6, 1978 amended in 2004, they had the right to access and rectify information concerning them.

## Author Contributions

SO, SR, and AS contributed to the preparation of the manuscript. SR and AS coordinated the project. SO collected the data. All authors listed have made substantial, direct, intellectual contribution to the work, and have approved it for publication.

## Conflict of Interest

The authors declare that the research was conducted in the absence of any commercial or financial relationships that could be construed as a potential conflict of interest.

## Publisher's Note

All claims expressed in this article are solely those of the authors and do not necessarily represent those of their affiliated organizations, or those of the publisher, the editors and the reviewers. Any product that may be evaluated in this article, or claim that may be made by its manufacturer, is not guaranteed or endorsed by the publisher.
